# Detoxification of Deoxynivalenol by a Mixed Culture of Soil Bacteria With 3*-epi-*Deoxynivalenol as the Main Intermediate

**DOI:** 10.3389/fmicb.2019.02172

**Published:** 2019-09-20

**Authors:** Yaoyao Zhai, Lei Zhong, Hui Gao, Zhaoxin Lu, Xiaomei Bie, Haizhen Zhao, Chong Zhang, Fengxia Lu

**Affiliations:** Laboratory of Enzyme Engineering, College of Food Science and Technology, Nanjing Agricultural University, Nanjing, China

**Keywords:** deoxynivalenol, enrichment, epimerization, bacterial consortium, mixed culture *Pseudomonas*, *Lysobacter*

## Abstract

Deoxynivalenol (DON) is a widely distributed mycotoxin that frequently occurs in various agricultural raw materials and feeds. DON acts as a virulence factor that accelerates the spread of plant diseases; moreover, its accumulation in grains causes yield loss and serious health problems to humans and livestock. Biodegradation of DON into less- or non-toxic substances using naturally existing microorganisms is considered the best approach for DON detoxification. Although various single isolates and mixed cultures capable of detoxifying DON have been reported, details of the metabolic pathways and the degrading enzymes/coding genes involved are scarce. In this study, we aimed to isolate DON-degrading bacteria from soil samples and explore the mechanisms. Toward this end, 85 soil samples collected from different provinces in China were enriched under aerobic conditions with mineral media containing 50 μg/ml of DON as the sole carbon source. The bacterial consortium LZ**-**N1 exhibited highly efficient and steady DON-transforming activity. High-throughput sequencing was used to characterize the composition of the involved microflora, and analysis of 16S rRNA sequences indicated that LZ**-**N1 was composed of at least 11 bacterial genera, with *Pseudomonas* accounting for nearly half the relative abundance. Coincubation of a mixed culture of two novel strains from the LZ-N1 consortium, namely *Pseudomonas* sp. Y1 and *Lysobacter* sp. S1, showed sustained transformation of DON into the metabolite 3-*epi*-deoxynivalenol, with no degradation products detected after 72 h. The cell-free supernatant, lysate, and cell debris of the mixed culture possessed DON-degrading ability, with the supernatant reaching a DON degradation rate of 100% within 48 h with 50 μg/ml of DON. This is the first report of two-step enzymatic epimerization of DON by a mixed culture, which may provide a new insight into this pathway for future applications in detoxification of DON-contaminated cereals and feed.

## Highlights

1. The mixed culture of *Pseudomonas* sp. Y1 and *Lysobacte*r sp. S1 with DON-degrading ability was isolated from soil samples.
2. The main intermediate metabolite was identified as 3-*epi*-deoxynivalenol.
3. The characteristics of DON epimerization proceeded by mixed culture were investigated.

## Introduction

Deoxynivalenol (3ɑ,7ɑ,15**-**trihydroxy**-**12,13**-**epoxytrichothec**-**9**-**en**-**8**-**one, DON), also referred to as vomitoxin, is a common trichothecene produced by many *Fusarium* species (He et al., 2009; Awad et al., 2010; van der Lee et al., 2015). DON not only threatens global food security (Volkl et al., 2004; Li et al., 2017) but also triggers an array of pathological responses in humans and animals, such as appetite disturbance, dyspeptic symptoms, and immune suppression (Pestka, 2007; Kolfclauw et al., 2009; Miller and Ewen, 2010; Sobrova et al., 2010). DON exerts its toxic effects through inhibition of eukaryotic protein synthesis by binding to the 60S subunit of the ribosome (Hassan et al., 2015). Owing to its remarkable chemical stability and high thermostability (170–350°C), conventional chemical/physical methods do not achieve effective DON decontamination (Jaukovic et al., 2014). Thus, a feasible and sustainable approach to address accidental DON contamination of food and feed commodities is urgently needed.

Among the multiple detoxification strategies, biodegradation by microorganism is undoubtedly a powerful approach for DON decontamination (Pierron et al., 2016; Tian et al., 2016; Lyu et al., 2018). The epoxide ring at C12**-**13 and the hydroxy group at C3 in DON are considered highly reactive and crucial for DON toxicity (Gowrinathan et al., 2011; Islam et al., 2012; Pierron et al., 2016; He et al., 2017). De-epoxidation of DON to de**-**epoxy**-**deoxynivalenol (DOM**-**1) under anaerobic/aerobic conditions by various microorganisms was reported in the last few decades; for instance, *Eubacterium* BBSH 797, *Bacillus* sp. LS100, and consortium C133; however, this mechanism is still unknown owing to the absence of information on the involved degrading enzymes/genes (Cote et al., 1986; He et al., 1992; Guan et al., 2009; Li et al., 2011; Ahad et al., 2017). Aerobic biotransformation processes of DON by targeting C3-OH, including oxidation and epimerization, which results in the formation of 3-keto-deoxynivalenol (3-keto-DON) by *Agrobacterium-Rhizobium* E3-39 or D107 (Shima et al., 1997; Fuchs et al., 2000; Wilson et al., 2017) and 3-*epi*-deoxynivalenol (3**-***epi***-**DON) by *Nocardioides* WSN05-2, *Devosia mutans* 17-2-E-8, and *Sphingomonas* S3-4, respectively (Ikunaga et al., 2011; Hassan et al., 2016; He et al., 2016a,b), have also been reported. Furthermore, glycosylation, acetylation, hydration, and hydroxylation have been identified to be involved in the process, based on decreased toxicity of the final metabolites compared with that of DON (Poppenberger et al., 2003; He et al., 2015; Pierron et al., 2016; Springler et al., 2017). Although DON-degrading microorganisms have been isolated, there is a lack of information on the involved metabolic pathways, enzymes, and genes due to cometabolism and volatile ability, thereby hampering the development of effective DON biotransformation strategies. Thus, characterization of DON-degrading microorganisms and investigation of degradation pathway are important to explore involved enzymes/genes for DON degradation.

Here, we have isolated two novel strains responsible for DON degradation, namely *Pseudomonas* sp. Y1 and *Lysobacter* sp. S1, from soil samples. A mixed culture of Y1 and S1 transformed DON into 3-*epi*-DON as the main intermediate metabolite, which is less toxic than DON. The DON epimerization by the mixed culture may be related to the cooperative metabolism between the two strains. This new finding provides a new perspective on the common detoxification pathway in soil bacteria and the mixed culture would be an effective biological agent for DON detoxification.

## Materials and Methods

### Chemicals and Media

DON/3-keto-DON standards were purchased from TripleBond (Guelph, Canada), solubilized in sterile water to bring the initial concentrations to 1.0 mg/ml, filtered with 0.22 μm membrane filters, and stored at 4°C until used. Water was purified by a Milli-Q water system (Millipore Corporation, New York, USA). Mineral salt medium (MM) was formulated as described previously (Ikunaga et al., 2011), but with slight modifications. In brief, the medium contained 1.6 g of Na_2_SO_4_, 1.0 g of KH_2_PO_4_, 0.5 g of MgSO_4_, 0.5 g of NaNO_3_, 0.5 g of (NH_4_)_2_SO_4_, 0.025 g of CaCl_2_, and DON as the carbon source. Mineral salt-based medium (MSB) was composed of basic MM supplemented with 10% peptone. Luria-Bertani (LB) medium contained 10.0 g of tryptone, 5.0 g of yeast extract, and 10.0 g of NaCl. HPLC-grade methanol and analytical-grade ethyl acetate were obtained from Sinopharm (Shanghai, China). Cycloheximide was purchased from Solarbio (Beijing, China).

### Apparatus and Conditions for High-Performance Liquid Chromatography and Preparative Liquid Chromatography

High-performance liquid chromatography (HPLC) analysis was conducted using an Ultimate 3000 station (Thermo, Shanghai, China) equipped with an ultraviolet detector. The separation was performed using a 5HC-C18 analytical column (250 mm × 4.6 mm, Agilent, CA, USA). Isocratic elution was performed with methanol and water (30:70, v/v) at a flow velocity of 0.6 ml/min. Twenty microliters of each sample was injected to the column for DON analysis. Preparative liquid chromatography (Pre-LC) was performed using a Waters 600 controller (Waters, Massachusetts, USA) equipped with an ultraviolet detector. The separation was performed using an XBridge C**-**18 column (19 mm × 150 mm, film thickness 5 μm; Waters, MC, USA). Isocratic elution was performed with methanol and water (15:85, v/v) at a flow velocity of 6 ml/min. Two milliliters of each sample were injected to the column for DON purification. HPLC and pre-LC were both performed at a wavelength of 218 nm.

### Enrichment of Deoxynivalenol-Degrading Bacteria

Eighty-five soil samples were collected from different locations of farmland and uncultivated soils at Anhui, Jiangsu, Henan, Hebei, Shanxi, and Hainan Provinces in China. Three out of 85 soil samples were enriched by adding 20 ml of DON solution (10 μg/ml) to the natural environment each month for 3 months. Approximately, 0.5 g of soil was suspended in 4 ml MM (50 μg/ml DON) and cultured at 30°C with shaking at 180 rpm for 1 week. To inhibit fungal growth, 100 μg/ml of cycloheximide was added to the culture. Next, 100 μl of the enriched culture was transferred to 4 ml of fresh medium and further incubated under the same condition for another 1 week. At least, three rounds of enrichment were performed. Soil sample autoclaved at 121°C for 20 min was used as a positive control to exclude physical adsorption. Enriched cultures with DON-degrading ability were centrifuged (8,228 ×*g*, 4°C, 10 min) and filtered through 0.22-μm membrane filters. DON concentrations were determined by HPLC.

### Analysis of Bacterial Population Diversity With Deoxynivalenol-Degrading Ability

Microbial DNA of consortium with DON-degrading ability was extracted using the FastDNA^®^ SPIN Kit (MP Biomedicals, CA, USA) according to manufacturer’s protocols. The final DNA concentration and purification were determined by NanoDrop 2000 UV-Vis spectrophotometer (Thermo Scientific, Wilmington, USA), and DNA quality was checked by 1% agarose gel electrophoresis. The V3-V4 hypervariable regions of the bacteria 16S rRNA gene were amplified with primers 338F (5′-ACTCCTACGGGAGGCAGCAG-3′) and 806R (5′-GGACTACHVGGGTWTCTAAT-3′) by thermocycler PCR system (GeneAmp 9700, ABI, USA). The PCR reactions were conducted using the following program: 3 min of denaturation at 95°C, 27 cycles of 30 s at 95°C, 30 s for annealing at 55°C, and 45 s for elongation at 72°C, and a final extension at 72°C for 10 min. PCR reactions were performed in triplicate 20 μl mixture containing 4 μl of 5× FastPfu Buffer, 2 μl of 2.5 mM dNTPs, 0.8 μl of each primer (5 μM), 0.4 μl of FastPfu Polymerase 0.2 μl of BSA, and 10 ng of template DNA. The resulted PCR products were extracted from a 2% agarose gel and further purified using the AxyPrep DNA Gel Extraction Kit (Axygen Biosciences, Union City, CA, USA) and quantified using QuantiFluor™-ST (Promega, USA) according to the manufacturer’s protocol.

Purified amplicons were pooled in equimolar and paired-end sequenced on an Illumina MiSeq platform (Illumina, San Diego, USA) according to the standard protocols by Majorbio Bio-Pharm Technology Co. Ltd. (Shanghai, China). Raw fastq files were quality-filtered by Trimmomatic and merged by FLASH. Operational taxonomic units (OTUs) were clustered with 97% similarity cutoff using UPARSE (version 7.1, http://drive5.com/uparse/) with a novel “greedy” algorithm that performs chimera filtering and OTU clustering simultaneously. The taxonomy of each 16S rRNA gene sequence was analyzed by RDP Classifier algorithm^1^ against the Silva (SSU123) 16S rRNA database using confidence threshold of 70%. Bacterial diversity was analyzed using an online cloud platform^2^.

### Isolation of Deoxynivalenol-Degrading Bacteria

To isolate pure strains, the consortium LZ-N1 that cultured in MM was serially diluted and approximately 100 μl of each dilution was spread on MM plates, 30°C for 1 week. All the colonies were selected and cultured in 1 ml fresh MM containing 10 μg/ml of DON at 30°C, shaking at 180 rpm for 1 week. DON degradation efficiency among different colonies was monitored by HPLC. Afterwards, MM, MSB, and LB plates were used to purify bacterial colonies.

### 16S rRNA Sequencing of Deoxynivalenol-Degrading Bacteria

The DON-degrading bacteria were characterized using 16S rRNA gene sequencing. Whole genome for PCR was prepared using a bacterial DNA extraction kit (D3350-01; Omega, Norcross, USA) according to the manufacturer’s instructions. The reaction mixture contained 25 μl of 2 × Taq Master Mix (P111-01; Vazyme, Nanjing, China,), 2 μl of each primer (10 mM), 1 μl of template DNA, and 19 μl of ddH_2_O. PCR was performed using an ABI PCR 2720 thermo-cycler (Nanjing, China) under the following cycling conditions: 95°C for 3 min; 30 cycles of 95°C for 30 s, 56°C for 30 s, and 72°C for 1 min 30 s; 72°C for 7 min; and a 4°C soak. The amplified products were sequenced by Sangon Biotech (Shanghai, China). Sequences showing similarity (>97%) to the 16S rRNA gene of isolated strains were identified using BLAST searches, and phylogenetic trees were constructed using the MEGA version 6.0 software (Tamura et al., 2013).

### Purification and Characterization of 3*-epi*-Deoxynivalenol

DON-degrading bacteria were cultivated in 50 ml MM containing 500 μg/ml of DON for 60 h and harvested by centrifugation (8,228 ×*g*, 4°C, 10 min). The supernatant was injected into the pre-LC column, and products of DON degradation were identified according to retention times and verified by HPLC. Collected fractions were concentrated using a rotary evaporator and dissolved in water. Then, the products extracted thrice each time using 5 ml of ethyl acetate. The upper layer was collected and dried using a nitrogen stream to remove ethyl acetate. Finally, 5.0 mg of pure compound A was obtained. The structure of compound A was determined by HQ-TOF-LC/MS/MS (AB Sciex Instruments, USA) and NMR spectrometer (Bruker, Germany).

### Quantification of Deoxynivalenol Degradation and Bacterial Growth

Bacteria cultured in MM containing 50 μg/ml of DON for 24 h were transferred to fresh MM containing 1% inoculum. Next, the OD_600_ values were measured with a UV-Vis spectrophotometer (Shimadzu, Japan), and the concentrations of DON and compound A, the main degradation product, were determined every 12 h by HPLC.

### Preparation of Supernatants, Cell Lysates, and Cell Debris of the Mixed Culture

DON transformation was examined by adding DON to culture supernatants, cell lysates, and cell debris of mixed culture of Y1 and S1. Briefly, the mixed culture was cultivated in MM for 72 h. To obtain lysates, the cells were harvested by centrifugation (8,228 ×*g*, 4°C, 10 min), washed twice with 50 mM of phosphate buffered solution (PBS) buffer (pH 7.2), suspended in PBS, and disintegrated by continuous ultrasonication for 30 min. The resulting supernatants were collected by centrifugation (12,857 ×*g*, 4°C, 30 min) as lysates, whereas the precipitates were suspended in PBS as cell debris. To remove bacterial cells, the culture supernatants and lysates were filtered through 0.22-μm membrane discs (Agela Technologies, Tianjin, China). Culture supernatants, cell lysates, and cell debris heated in boiling water for 30 min were considered heat-inactivated samples. To examine DON-degrading ability, DON was added to the culture supernatant, cell lysates, and cell debris to a final concentration of 50 μg/ml, and the mixtures were placed in a thermostatic chamber (Shanghai Sumsung Laboratory Instrument Co., Ltd., Shanghai, China) at 30°C. Reactions were stopped by adding acidified methanol and filtration through 0.22-μm membrane filters. Changes in DON and 3-*epi*-DON concentrations at different time points were monitored by HPLC.

### Analysis of Deoxynivalenol, 3-keto-Deoxynivalenol, and 3-*epi*-Deoxynivalenol

For analysis of DON, 3-keto-DON, and 3-*epi*-DON in the bacterial epimerization pathway, the mixed culture was coincubated with DON or 3-keto-DON as a substrate. The mixed culture was cultivated in MM for 72 h, and DON and 3-keto-DON were added to the mixed culture at 30°C. Reactions were stopped by adding acidified methanol. Next, the culture was filtered through 0.22-μm membrane filters. Changes in DON, 3-keto-DON, and 3-*epi*-DON concentrations at different time points were monitored by HPLC.

### Statistical Analysis

All tests were performed in triplicate, and the values presented are the mean of three independent experiments ± standard deviation (SD) of the triplicate experiments. Excel 2013 was used to conduct the statistical analyses (Microsoft, Redmond, Washington, USA).

## Results

### Deoxynivalenol Detoxification by Soil Samples

**Figure 1:**
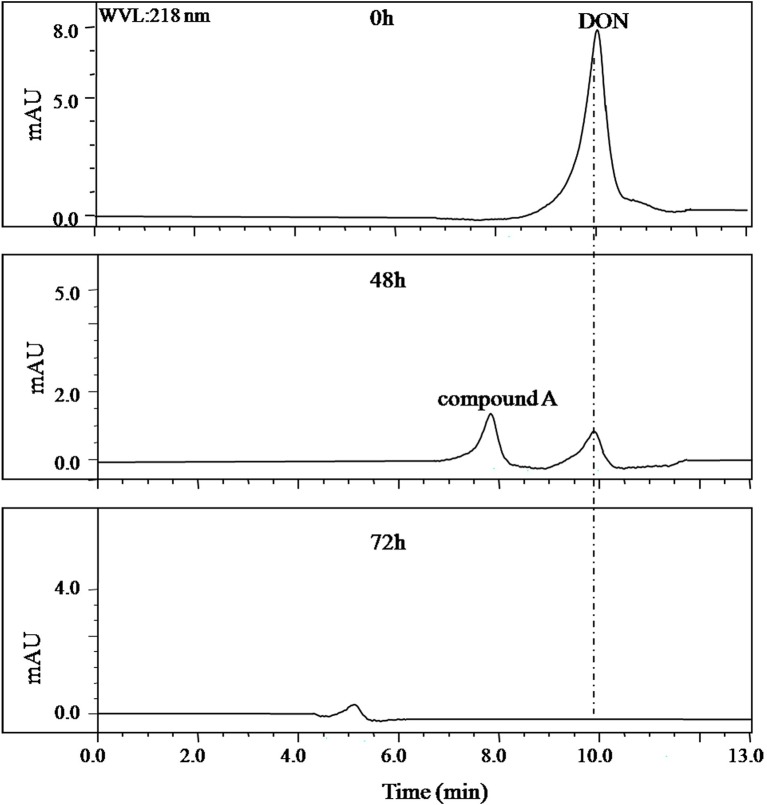
HPLC profile of the culture medium of the bacterial consortium LZ-N1. The bacterial consortium was cultured in MM (containing 50 μg/ml DON), and the peak area of DON in different time points (0, 48, and 72 h) was detected by HPLC.

In the collected samples, the one plant rhizosphere soil from Nanjing that collected after DON enrichment under natural environment showed the stability in DON degradation after several subcultures at laboratory conditions. This consortium contained in this soil sample was later named as LZ-N1. The batch degradation experiments showed that 50 μg/ml of DON was completely degraded by the bacterial consortium LZ-N1 within 72 h (Figure 1).

### Composition of the Bacterial Consortium

**Figure 2:**
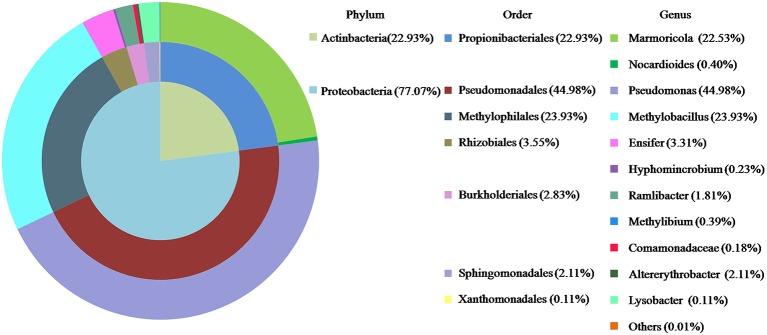
Bacterial diversity of LZ-N1. In the colorful pie chart, components are classified according to phylum, order, and genus from inside to outside, and presented with different colors.

The composition of LZ-N1 and its bacterial diversity are presented (Figure 2). All operational taxonomic units belonged to the phyla *Proteobacteria* (77.07%) and *Actinobacteria* (22.93%). A total of seven orders were identified—*Pseudomonadales* (44.98%), *Methylophilales* (23.93%), *Propionibacteriales* (22.93%), *Rhizobiales* (3.55%), *Burkholderiales* (2.38%), *Sphingomonadales* (2.11%), and *Xanthimonadales* (0.11%). Eleven genera accounted for 99.99% of the sequences, and *Pseudomonas* had the highest relative abundance (accounting for 44.98% of the sequences). The population structure of LZ-N1 bacterial consortium differed from the previously reported ones, which was able to effectively biodegrade DON into 3-epi-DON under aerobic condition, including the genera *Acinetobacter*, *Leadbetterella*, and *Gemmata* (Wilson et al., 2017). The raw reads of the consortium LZ-N1 were deposited into the NCBI Sequence Read Archive database (Accession Number: PRJNA557441; https://www.ncbi.nlm.nih.gov/bioproject/PRJNA557441).

### 16S rRNA Sequences Analysis of Deoxynivalenol-Degrading Bacteria

**Figure 3:**
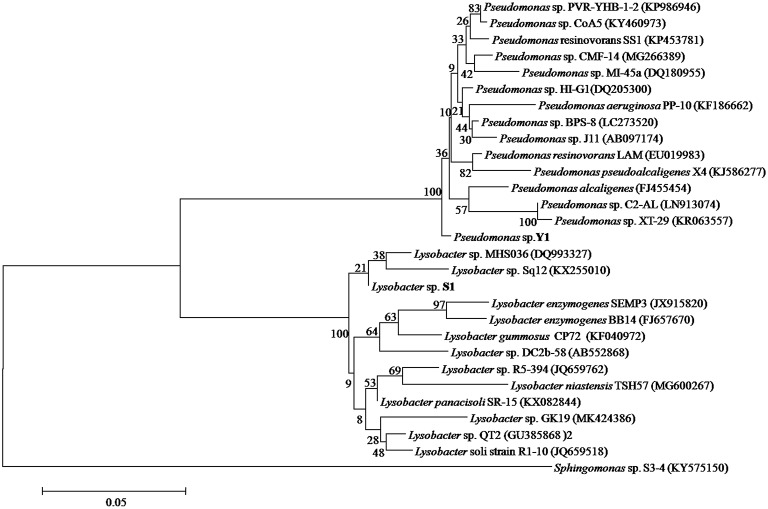
Phylogenetic tree of *Pseudomonas* sp. Y1 and *Lysobacter* sp. S1. The 16S rRNA sequences of Y1 and S1 were aligned using BLAST tool and the Neighbor-Joining Tree was constructed using MEGA 6.0.

A total of 357 colonies were selected from serially diluted MM (containing 10 μg/ml of DON) plates and tested for DON-degrading ability. Five colonies showed varying degradation capacity. Results of separation using plate streaking method in MM, MSB, and LB medium showed that these colonies comprised of more than one strain, and the *Pseudomonas* species was the common microorganism, without exception. However, the *Pseudomonas* species from these colonies showed no DON-degrading ability when grown on MM medium. A mixed culture of two strains found in one colony, which were designated as Y1 and S1, degraded DON completely. In 16S rRNA gene analysis, the 1,369 bp of strain Y1 and 1,441 bp of strain S1 showed 98.46 and 97.54% identity to the sequences of *Pseudomonas alcaligenes* and *Lysobacter enzymogenes* SEMP3, respectively. The neighbor-joining phylogenetic tree (Figure 3) based on the partial 16S rRNA gene sequence also suggested that Y1 was closely related to the genus *Pseudomonas* and S1 to the genus *Lysobacter*. The 16S rRNA sequence of Y1 and S1 were deposited into the NCBI GenBank database (Accession Number: MN197750 and MN197751, respectively).

### Purification and Identification of the Intermediate 3-*epi*-Deoxynivalenol

**Figure 4:**
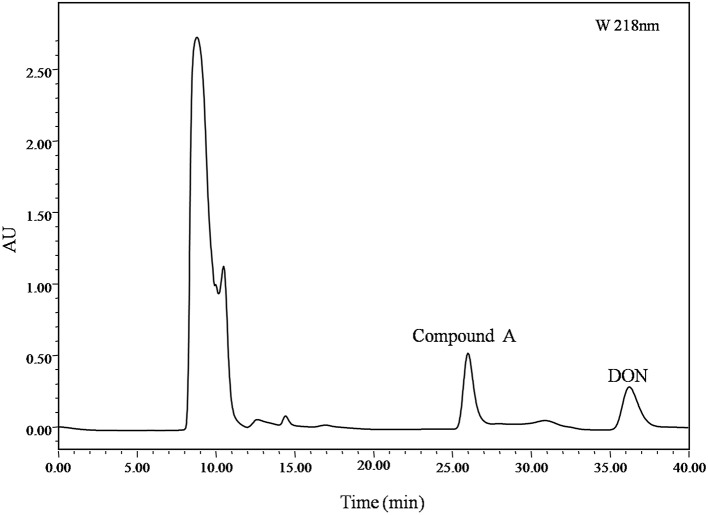
pre-LC profile of fermented supernatant cultured with mixed culture of *Pseudomonas* sp. Y1 and *Lysobacter* sp. S1. The mixed culture of Y1 and S1 was cultivated in 50 ml MM (containing 500 μg/ml DON) for 72 h, and the fermented supernatant was obtained by centrifugation (8,228 ×*g*, 4°C, 10 min) to remove bacterial cells. Compound A was eluted at 26.0 min, separated, and purified with pre-LC.

Identification of DON degradation product is essential for determining the involved pathways. Mixed cultures of Y1 and S1 synergistically transformed DON. Through pre-LC followed by analytical HPLC, one major intermediate, compound A, was revealed at 26.0 and 8.0 min of elution (Figures 1, 4), respectively.

**Figure 5:**
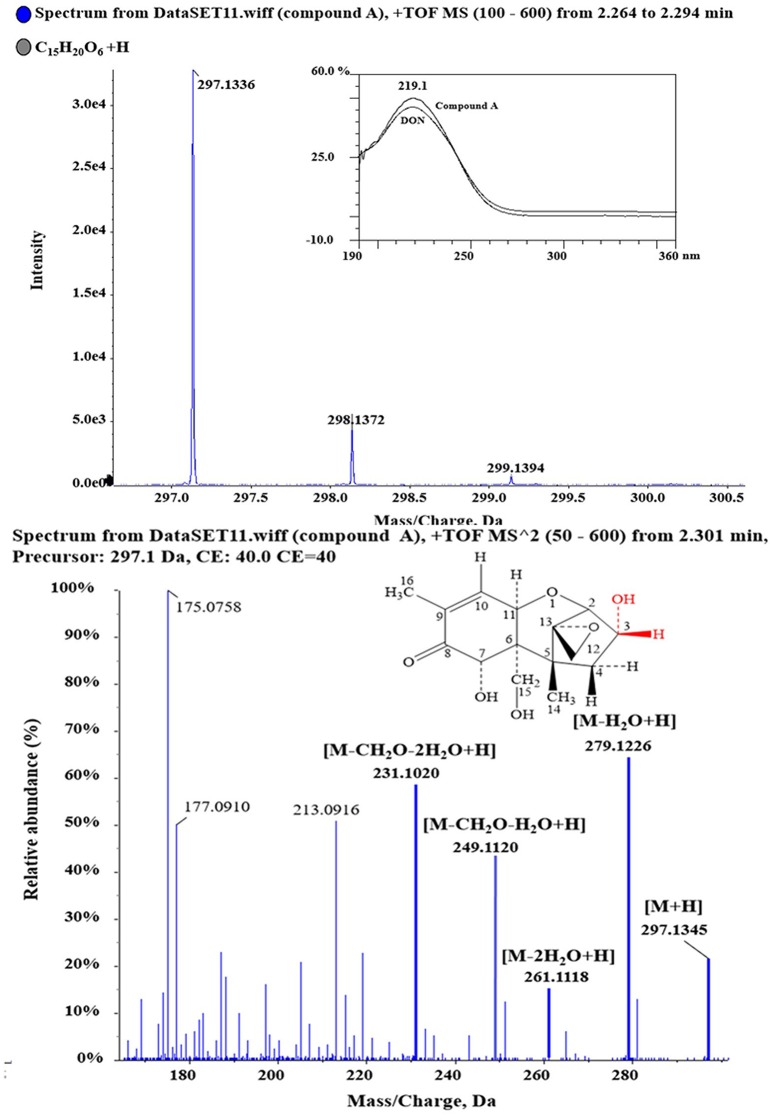
1D and 2D MS spectra of compound A. Purified compound A was analyzed with HQ-TOF-LC/MS/MS, and the 1D and 2D MS spectra were obtained.

Purified compound A (5 mg) was fully characterized by Hybrid Quadruple-TOF LC/MS/MS and ^1^H and ^13^C NMR spectroscopy. The molecular ion peak was at 297.1336 m/z, and the formula was C_15_H_20_O_6_ (Figure 5). The characteristic ions of compound A at 397, 279, 261, 249, and 231 were the same as those of DON, but their relative abundances differed (He, 2015). DON and compound A had spectral maxima at approximately 220 nm, and MS and NMR data revealed that the mixed culture of Y1 and S1 epimerized the C3-OH of DON (Table 1). The 3-*epi*-DON proton data closely resembled that reported previously (Ikunaga et al., 2011; Sato et al., 2012; Hassan et al., 2017; He et al., 2017).

**Table 1:** NMR data of DON and compound A, obtained using a Bruker Avance-600 spectrometer (in CDCl_3_ at 25°C).

Position	^1^H-NMR δ_H_, ppm (mult., J in Hz)		^13^C-NMR δ_C_ (ppm)	
	DON	Compound A	DON	Compound A
**2**	3.65 (d, 4.4)	3.80 (s)	80.8	84.1
**3**	4.55 (dt, 4.4, 4.4, 10.4)	4.36 (d, 7.4)	69.2	72.5
**4**	2.23 (dd, 4.0, 14.4)	2.96 (dd, 7.6, 15.3)	43.2	46.2
	2.10 (dd, 10.8, 14.8)	1.60 (s)		
**5**			46.5	46.2
**6**			52.0	51.8
**7**	4.83 (s)	4.78 (s)	74.5	74.0
**8**			199.9	200.0
**9**			136.0	136.3
**10**	6.63 (dd, 1.3, 5.8)	6.57 (dd, 1.5, 5.8)	138.5	138.1
**11**	4.55 (dt, 4.4, 10.8)	4.21 (d, 5.8)	70.4	70.1
**12**			65.6	65.4
**13**	3.10 (d, 4.2)	3.19 (d, 4.2)	47.4	47.2
	3.18 (d, 4.3)	3.14 (d, 4.2)		
**14**	1.15 (s)	1.25 (d, 4.3)	14.3	14.1
**15**	3.91 (d, 11.7)	3.84 (d, 11.7)	62.5	62.1
	3.76 (d, 11.7)	3.74 (d,11.1)		
**16**	1.91 (s)	1.89 (s)	15.4	15.3

*δ_H_ and δ_C_ represent chemical shift of H and C atom in standard deoxynivalenol (DON) and compound A*.

### Characteristics of Deoxynivalenol Degradation by the Mixed Culture

**Figure 6:**
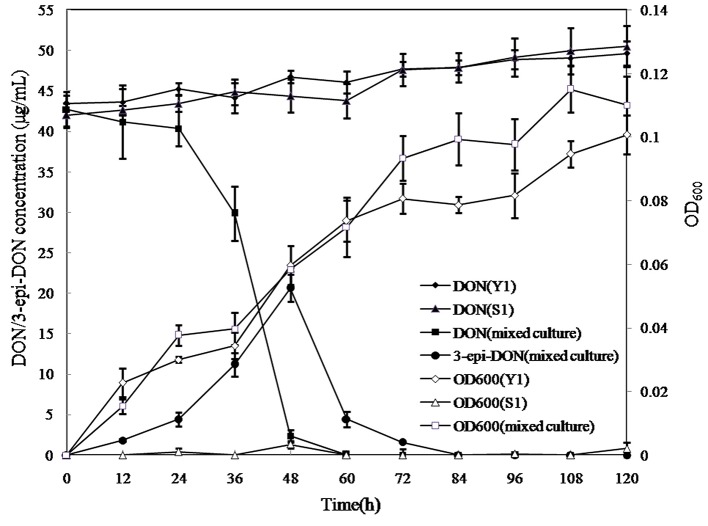
DON degradation and bacterial growth profiles of *Pseudomonas* sp. Y1, *Lysobacter* sp. S1, and the mixed cultures. Different curves represent changes in DON concentration or the OD_600_ values of bacterial cultures. DON (Y1) represents DON concentration in MM cultured with strain Y1; DON (S1) represents DON concentration in MM cultured with strain S1; DON (mixed culture) represents DON concentration in MM cultured with the mixed culture of Y1 and S1; 3-*epi*-DON (mixed culture) represents 3-*epi*-DON concentration in MM cultured with the mixed culture of Y1 and S1; OD600 (Y1) represents the OD_600_ values of Y1 culture; S1 represents the OD_600_ values of S1 culture; (mixed culture) represent the OD_600_ values of the mixed culture of Y1 and S1.

In the mixed culture, DON disappeared completely within 60 h, whereas 3-*epi*-DON level increased up to 48 h and then decreased until disappearing completely at 84 h, indicating that 3-*epi*-DON was not the final product (Figure 6). Interestingly, Y1 or S1 showed no DON-degrading activity only when grown on MM, although Y1 thrived on MM containing DON. This result indicated that the DON degradation process involved a cooperative mechanism between Y1 and S1.

**Figure 7:**
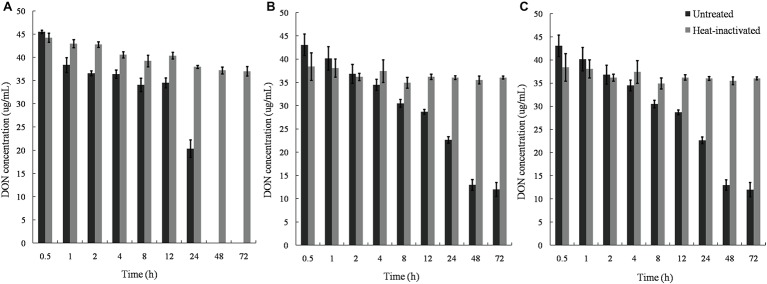
DON degradation by the culture supernatant, cell lysates, and cell debris of *Pseudomonas* sp. Y1 and *Lysobacter* sp. S1. **(A)** DON degradation by culture supernatant in different time points; **(B)** DON degradation by lysate in different time points; **(C)** DON degradation by cell debris in different time points at 30°C.

Untreated supernatants of the mixed culture degraded DON completely within 48 h, whereas the cell debris and lysates degraded approximately 50% DON in 24 and 8 h, with no further change in DON concentration after 48 and 12 h. In contrast, heat-inactivated supernatant, lysate, and cell debris did not show any DON-degrading ability, indicating that enzymatic reactions were involved in the DON-degrading ability of the mixed culture (Figure 7).

### Deoxynivalenol Epimerization in the Mixed Culture

**Figure 8:**
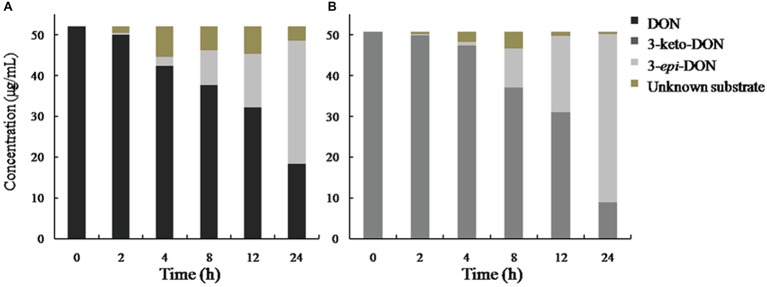
DON and 3-keto-DON transformation by a mixed culture of *Pseudomonas* sp. Y1 and *Lysobacter* sp. S1. DON or 3-keto-DON was added to whole-cell mixed culture of Y1 and S1, and the concentrations of DON, 3-keto-DON, and 3-*epi*-DON were measured by HPLC. **(A)** DON as a substrate; **(B)** 3-keto-DON as a substrate.

In our experiment, the mixed culture showed bacterial epimerization as a route of DON detoxification under aerobic condition. The mixed culture converted DON and 3-keto-DON into 3-*epi*-DON within 24 h, showing no 3-keto-DON accumulation when DON included as a substrate in the medium (Figure 8).

## Discussion

The composition of LZ-N1 obtained in our study differed widely from that of other reported DON-detoxification consortia (Volkl et al., 2004; Yoshizawa et al., 2006; Ahad et al., 2017; Wilson et al., 2017). Many previously reported genera with DON-degrading ability were also found in LZ-N1. For instance, *Nocardioides* isolated from a wheat field transforms DON into 3-*epi*-DON (Ikunaga et al., 2011; Sato et al., 2012), and *Marmoricola* MIM116 derived from wheat head degrades DON to an unknown metabolite (Ito et al., 2012). *Marmoricola* and *Nocardioides* accounted for 22.53 and 0.40% relative abundance of LZ-N1, respectively, but no strains belonging to these genera were isolated. *Pseudomonas* species also found in another bacterial consortium PCG-3 with DON de-epoxidation ability derived from soil samples, while *Lysobacter* species were barely reported as DON-detoxification bacteria (He et al., 2016a,b). Thus, we speculated that the DON metabolic pathway of LZ-N1 was different from that of previously reported DON-degrading organisms.

DON-degrading bacteria are widespread in various natural environments, but isolation of active pure cultures of these bacteria is challenging owing to factors such as coexistence with dominant non-DON-degrading microorganisms, non-favorable laboratory culture conditions, and instability of induced DON-degrading activity (He, 2015). Furthermore, the process of DON detoxification was achieved *via* cometabolism, necessitating the participation of multiple microorganisms. Inferior strains were usually considered as DON-degrading bacteria when DON was used as the sole carbon source in MM media; however, some dominant strains were less focused, especially the multiple reactions involved degradation processes. In this study, the mixed culture of relatively dominant *Pseudomonas* sp. Y1 (44.98%) and inferior strain *Lysobacter* sp. S1 (0.11%) showed superior ability in DON degradation. In the present study, Y1 and S1 were found to be responsible for the DON-detoxifying effect of the bacterial consortium, indicating the existence of a collaborative metabolism between these two strains. In this type of metabolism, one of the isolates possibly produces the enzymes required to achieve the first-step conversion of DON (a key step), whereas the other utilizes the intermediate produced in this first step as a carbon source for growth and supplies necessary nutrients/energy to its counterpart. Genomics (including next**-**generation sequencing), proteomics, and computational biology may provide powerful approaches for investigating unknown DON-degrading synergistic pathways. For instance, the identification of the aldo-keto reductase AKR18A1 from *Sphingomonas* S3-4, responsible for DON oxidation in C3-OH group, was based on the comparative analysis of its genome sequence with DON-degrading strain *Devosia mutans* 17-2-E-8, and non-DON-degrading strain *Sphingobium* S26, combined with functional screening of the S3-4 genomic BAC library (He et al., 2017). Based on the gene/protein sequences of several DON-degrading enzymes, PCR amplification and gene expression assisted verification and identification of DON-degrading genes/enzymes from newly identified bacteria could an effective alternative in revealing the DON degradation mechanism. The identification of relative genes/enzymes in DON-degradation by the strain *Pseudomonas* sp. Y1 and *Lysobacter* sp. S1 is still on-going.

A mixed culture of two soil bacteria was capable of transforming DON into the non-toxic 3-*epi*-DON. Compared with the heat-inactivated ones, the untreated cell-free supernatant of the mixed culture showed a high efficiency in DON degradation. It could conclude that DON degradation by *Pseudomonas* sp. Y1 and *Lysobacter* sp. S1 was an enzymatic epimerization process. To further explore the DON degradation pathway, DON and 3-keto-DONwere used as substrate and reacted with the mixed culture respectively, which 3-*epi*-DON was detected in the both reaction mixtures. Results showed that DON epimerization is a two-step enzymatic DON detoxification pathway that is considered common among soil microorganisms (both Gram-negative and Gram-positive), and it comprises of DON oxidation to 3-keto-DON, followed by selective reduction of 3-keto-DON to 3-*epi*-DON (Sato et al., 2012; Hassan et al., 2017; He et al., 2017). This metabolic pathway recently proven through chemical synthesis of 3-keto-DON and identification of the enzyme involved in 3**-**keto**-**DON accumulation in *Devosia mutans* 17-2-E-8 *via* two-step epimerization of DON (Carere et al., 2017, 2018; Hassan et al., 2017). Other reports have also shown that 3-keto-DON is the main intermediate for 3-*epi*-DON accumulation in many other *Devosia* species/strains (Hassan and Zhou, 2018). In our result, no accumulation of 3-keto-DON was observed, although the mixed culture transformed 3-keto-DON into 3-*epi*-DON, thereby suggesting that the epimerization mechanism was a consecutive two-step enzymatic reaction through formation of 3-keto-DON, but slightly different from that in the previous report (Hassan et al., 2017). A possible reason for this difference was the cometabolism by Y1 and S1 in the DON epimerization process. In general, the DON metabolic pathway in the mixed culture of Y1 and S1 differed from that in 17**-**2**-**E**-**8. First, the mixed culture was capable of assimilating DON as a carbon source, whereas 17**-**2**-**E**-**8 is not. Second, the biotransformation activity of 17**-**2**-**E**-**8 was exerted *via* DepA and DepB, which are intracellular dehydrogenases/oxidoreductases that use PQQ/NADPH as cofactors, whereas the cell-free culture supernatants of the mixed culture of Y1 and S1 achieved DON epimerization, indicating the involvement of an extracellular enzyme. Third, although epimerization by the mixed culture was also a two-step enzymatic reaction, it did not cause 3-keto-DON accumulation, unlike 17**-**2**-**E**-**8-mediated transformation. Our results enriched the pool of DON-degrading microorganisms and, contributed to revealing the enzymatic epimerization of DON by the mixed culture of *Pseudomonas* sp. Y1 and *Lysobacter* sp. S1 in the meanwhile. Although the specific enzymes involved in the DON biotransformation activity of the mixed culture could not be identified in this study, details of the DON metabolic mechanism warrant further investigation.

## Conclusion

Two soil-derived bacteria *Pseudomonas* sp. Y1 and *Lysobacter* sp. S1 were successfully isolated by *in situ* enrichment procedure, which exhibited DON-degrading ability in a liquid culture setting. The 3-epi-DON, considerably less toxic than DON, was identified as the main intermediate during this DON degradation. Moreover, it is highly possible that DON was enzymatically transformed by the mixed culture of strain Y1 and S1. Therefore, the mixed culture has great potential in degradation of DON-contaminated animal feeds.

## Data Availability Statement

The raw data supporting the conclusions of this manuscript will be made available by the authors, without undue reservation, to any qualified researcher.

## Author Contributions

FL, ZL, and YZ conceived the project. YZ, LZ, and HG designed and carried out the experiments. XB, HZ, and CZ provided suggestions during the experiments. YZ prepared the manuscript. YZ, LZ, HG, and FL contributed to the final version of the paper. All authors read and approved the final manuscript.

### Conflict of Interest

The authors declare that the research was conducted in the absence of any commercial or financial relationships that could be construed as a potential conflict of interest.

## Footnotes

^1^http://rdp.cme.msu.edu/

^2^www.i-sanger.com
